# The Genome of the Margined White Butterfly (*Pieris macdunnoughii*): Sex Chromosome Insights and the Power of Polishing with PoolSeq Data

**DOI:** 10.1093/gbe/evab053

**Published:** 2021-03-19

**Authors:** Rachel A Steward, Yu Okamura, Carol L Boggs, Heiko Vogel, Christopher W Wheat

**Affiliations:** 1 Department of Zoology, Stockholm University, Sweden; 2 Department of Insect Symbiosis, Max Planck Institute for Chemical Ecology, Jena, Germany; 3 School of the Earth, Ocean and Environment, University of South Carolina, Columbia, South Carolina, USA; 4 Department of Biology, University of South Carolina, Columbia, South Carolina, USA; 5 Rocky Mountain Biological Laboratory, Crested Butte, Colorado, USA

**Keywords:** genome, long-read sequencing, polishing, PoolSeq, *Pieris*, evolutionary trap

## Abstract

We report a chromosome-level assembly for *Pieris macdunnoughii*, a North American butterfly whose involvement in an evolutionary trap imposed by an invasive Eurasian mustard has made it an emerging model system for studying maladaptation in plant–insect interactions. Assembled using nearly 100× coverage of Oxford Nanopore long reads, the contig-level assembly comprised 106 contigs totaling 316,549,294 bases, with an N50 of 5.2 Mb. We polished the assembly with PoolSeq Illumina short-read data, demonstrating for the first time the comparable performance of individual and pooled short reads as polishing data sets. Extensive synteny between the reported contig-level assembly and a published, chromosome-level assembly of the European butterfly *Pieris napi* allowed us to generate a pseudochromosomal assembly of 47 contigs, placing 91.1% of our 317 Mb genome into a chromosomal framework. Additionally, we found support for a Z chromosome arrangement in *P. napi*, showing that the fusion event leading to this rearrangement predates the split between European and North American lineages of *Pieris* butterflies. This genome assembly and its functional annotation lay the groundwork for future research into the genetic basis of adaptive and maladaptive egg-laying behavior by *P. macdunnoughii*, contributing to our understanding of the susceptibility and responses of insects to evolutionary traps.

Significance

The North American butterfly *Pieris macdunnoughii* lays eggs on an invasive host plant that is lethal to its larvae and is emerging as a model system for the study of maladaptive responses to rapid environmental change. We constructed a high-quality genome for this butterfly using Nanopore long-read data. We further demonstrated the performance of pooled short-read data for genome polishing. This study demonstrates the performance of Nanopore-only assembly approaches, reveals a novel role for pooled sequencing data in genome assembly, and provides an important resource for advancing research on maladaptive plant–insect interactions.

## Introduction

Anthropogenically induced rapid environmental change has led to many novel interactions between species and their biotic and abiotic environments. In some cases, historically adaptive behaviors have become evolutionary traps, wherein species continue to respond to cues that are no longer reliably linked to beneficial outcomes (Schlaepfer et al. 2002; Robertson et al. 2013). Thus, organisms might mistime life history events (van Dyck et al. 2015), fail to adjust to novel predators (Brown et al. 2018), or use poor, novel resources despite good, historical alternatives being available (Szaz et al. 2015). Identifying genetic variation involved in evolutionary traps can uncover how such maladaptive interactions arise, evolve, or persist through time, potentially informing conservation management (Supple and Shapiro 2018; Robertson and Blumstein 2019). In many cases, however, the tools needed to begin these investigations are missing; notably, high-quality genome assemblies.

*Pieris macdunnoughii* is one of at least five species (or semispecies) of North American *Pieris* butterflies (Chew and Watt 2006), resulting from the holarctic expansion of the *Pieris napi* species complex into North America 3–5 Ma (Geiger and Shapiro 1992). This montane butterfly ranges across the southern Rocky Mountains and is a hostplant specialist, laying eggs and feeding upon plants in the Brassicaceae. Importantly, it is the focus of ongoing research into how specialization has resulted in maladaptive interactions with invasive Eurasian mustards, wherein females oviposit on invasive plants despite their lethality to caterpillars (Chew 1975, 1977; Nakajima et al. 2013; Nakajima and Boggs 2015; Steward and Boggs 2020; Steward et al. 2019). Given the extensive literature investigating interactions between *Pieris* species and their hostplants, and advances in understand these interactions at the genomic level (Edger et al. 2015), developing a functional genomics understanding of these interactions in a species, such as *P. macdunnoughii* is a logical next step, but one that is hindered by the lack of genomic resources for this species.

Here, we present an annotated chromosome-level genome for *P. macdunnoughii*, generated using high-coverage Oxford Nanopore Technology (ONT) data. In addition, we explore assembly pipelines that leverage data sets often used for nonmodel organisms, such as PoolSeq data (i.e., a combination of individuals in a single sequencing library). Long-read genomes generally suffer from low accuracy and benefit from polishing with short reads (Walker et al. 2014; Watson and Warr 2019). We compared improvements to the assembly when polished with individual versus PoolSeq short read data, as PoolSeq data can also serve as an initial data set quantifying genetic diversity within a population, greatly enriching the output of genome sequencing pipelines.

Finally, the recent publication of a chromosome-level genome for *P. napi* revealed unanticipated and extensive rearrangements compared with other Lepidoptera, despite no change in chromosomal count (Hill et al. 2019). Other high-quality genomes from *Pieris* are needed to discern whether the reciprocal translocation events leading to this rearrangement continue to shape the evolution of this lineage. Additionally, although the Z chromosome for *P. napi* was not completely assembled, it appears to have a novel fusion event (Hill et al. 2019; Pruisscher et al. 2021). By comparing our *P. macdunnoughii* assembly with the published *P. napi* genome, we shed light on evolutionary dynamics.

## Materials and Methods

### Genome Sequencing

*Pieris macdunnoughii* individuals were collected near Gothic, CO, USA. The thorax of one female, collected in 2014, was sampled for genome sequencing. High molecular weight genomic DNA was isolated from the sample using paramagnetic nanodiscs (Nanobind Tissue Big DNA kit, Circulomics). The isolated DNA was processed using the Short Read Eliminator XS (Circulomics), selectively precipitating high molecular weight DNA based on polyethylene glycol (PEG) and sodium chloride concentrations. Isolated DNA was sequenced on a MinION platform, with fast base-calling using GUPPY (v.3.2.10) in the miniKNOW (v.3.6.5) software. A total of 3.82 million reads (28.17 Gb) were generated from one flow cell (∼90× coverage). The N50 length of the fast base-called subreads was 17.24 kb. We repeated base-calling with the high-accuracy option in GUPPY (v.4.0.11) generating another set of raw reads with an N50 of 17.24 kb. Raw reads have been deposited (ENA accession: ERS5472768).

### Assembly

From the raw reads we generated three assemblies, fast base-calling assembled with Flye (v.2.7, Kolmogorov et al. 2019), high-accuracy base-calling assembled with Flye and high-accuracy base-calling assembled with NECAT (v.0.0.1, Chen et al. 2021). For both Flye and NECAT, genome size was set as 300mb. Flye was run with the -meta option, which improves assemblies with biased read coverage (i.e., mitochondrial genome), and two polishing iterations. We ran four rounds of Racon (v.1.4.13, Vaser et al. 2017) on each assembly, followed by Medaka (v.1.0.3, https://nanoporetech.github.io/medaka/, last accessed March 18, 2021) to polish the assemblies with the nanopore data, using default settings (Latorre-Pérez et al. 2020).

We consolidated haplotype redundancies in each assembly using PURGEhaplotigs (v.1.0.3, default settings, Roach et al. 2018). Quickmerge (v.0.3, Chakraborty et al. 2016) was used to merge the polished and purged Flye (self_assembly, -l 197436) and NECAT (hybrid_assembly) assemblies. Finally, we used HaploMerger2 (v.20180603, Huang et al. 2017), to collapse duplicated haploid content in the Quickmerge assembly. Quality and completeness of these preliminary genome assemblies were assessed using SeqKit (v.0.12.1, Shen et al. 2016) and BUSCO using lepidoptera_odb10 (v.4.1.2, Seppey et al. 2019). BUSCO scores were visualized in R with scripts modified from the BUSCO output. All work in R was supported by the *tidyverse* (v.1.3.0, Wickham et al. 2019) and *ggpubr* (v.0.4.0, Kassambara 2020) packages.

### Illumina Sequencing and Mapping

We produced two sets of Illumina short-read sequence data that were used to polish the draft assembly. The first set came from an individual *P. macdunnoughii* female collected in the Upper East River Valley, CO, USA in 2015. The second set came from pooled DNA (PoolSeq) from 18 adult *P. macdunnoughii* females reared in the lab in 2005. This lab population originated from the Upper East River Valley, but was likely more inbred than wild *P. macdunnoughii*. Extraction was per individual, using a commercial kit (cell and tissue DNA kit) for robotic extraction on a KingFisher Duo Prime purifier (ThermoFisher Scientific), following standard protocols with added RNAse A to remove RNA contamination. DNA concentration and purity were quantified using a Qubit 2.0 fluorometer (ThermoFisher Scientific) and nanodrop (ThermoFisher Scientific) and run on a 2% agarose gel stained with GelRed to visually ascertain that DNA fragmentation was minimal. Samples were then pooled using an equal amount of DNA from each individual and used for library preparation and sequencing (Illumina HiSeq), which was performed by SciLifeLab (Stockholm, Sweden), using 150-bp paired-end reads with 350 bp insert size.

Illumina sequencing data sets were filtered for PCR clones (Stacks 1.21; supplementary table S3, Supplementary Material online), adapters trimmed using truseq and nextera reference databases, and filtered for read quality (bbmap 34.86, bbduk2.sh) with a base quality threshold of 20 and a minimum read length of 40. We mapped reads to the v0.08 draft assembly with NextGenMap (supplementary table S4, Supplementary Material online, v.0.5.5, Sedlazeck et al. 2013) and assessed mapped reads using GoLeft (v.0.2.1, https://github.com/brentp/goleft/releases, last accessed March 18, 2021) and Qualimap (v.2.2.1, Okonechnikov et al. 2016).

### Short-read Polishing and Annotation Assessment

We polished the final unpolished genome with each set of the mapped Illumina short reads using Pilon (v.1.23, Walker et al. 2014). We compared the polished and unpolished genomes using SeqKit and BUSCO analyses. Higher read coverage can improve the quality of polishing by Pilon (Walker et al. 2014), so we used SeqKit to subsample 1.60 × 10^8^ of the individual sample reads. Polishing the draft genome with the subsample (coverage comparable to the PoolSeq data) had little impact when evaluated using BUSCOs (supplementary table S1, Supplementary Material online).

We used the Braker2 automated (v.2.1.5, Lomsadze et al. 2005; Stanke et al. 2006, 2008; Ter-Hovhannisyan et al. 2008; Buchfink et al. 2015; Hoff et al. 2016, 2019; Brůna et al. 2021) pipeline to generate comprehensive annotations of all three assemblies (v0.08, v0.09, v0.10). Prior to running Braker2 we soft-masked the assembly (supplementary tables S1 and S2, Supplementary Material online) with RED (v.05/22/2015, Girgis 2015) using the redmask.py wrapper (v0.0.2, https://github.com/nextgenusfs/redmask, last accessed March 18, 2021). We ran Braker2 in the genome and protein mode, using reference proteins from the Arthropoda section of OrthoDB (v.10). We also ran EggNOG on the final PoolSeq-polished genome annotation to generate a functional annotation.

Annotations were assessed using the longest ortholog hit ratio (OHR), modified from O'Neil et al. (2010) for proteins. For each of the draft annotations (v0.08, v0.09, v0.10), we used CD-HIT (v.4.8.1, Li and Godzik 2006; Fu et al. 2012) to collapse protein clusters, from which we created blast databases (NCBI BLAST v. 2.5.0). The *Bombyx mori* protein set was accessed from NCBI (GCF_000151625.1_ASM15162v1). Each *B. mori* protein was blasted against the *P. macdunnoughii* databases. We calculated OHR as the proportion of a *B. mori* protein that was overlapped by an orthologous alignment hit in the draft *P. macdunnoughii* annotations. In our analysis, we focus on the OHR of the longest overlapping sequence.

### Synteny and Pseudochromosomal Assembly

We used *nucmer* (MUMmer4, v.4.0.0beta2, Marçais et al. 2018) to align the PoolSeq-polished *P. macdunnoughii* genome to the genome of the closely related Eurasian species *P. napi* (Hill et al. 2019). Due to considerable synteny between the two *Pieris* genomes, we used the *P. napi* genome as a reference to arrange the PoolSeq-polished *P. macdunnoughii* scaffolds into putative chromosomes for a pseudochromosomal assembly. To do this, RagTag (Alonge et al. 2019) was used to group and orient scaffolds against chromosomes 1–25 of the *P. napi* genome. We excluded unplaced *P. napi* scaffolds to target chromosomal placements of the *P. macdunnoughii* contigs, setting a lower grouping confidence threshold of 80% to avoid chimeric chromosomes. RagTag added 100 N between each joined scaffold, increasing the length of the genome by 5,600 bp. Synteny was visualized in R with the packages *circlize* (v 0.4.12, Gu 2014) and *RColorBrewer* (v.1.1-2, Neuwirth 2014).

### Genome-wide Variation

To illustrate their potential for subsequent population genetic inferences, the PoolSeq reads were mapped to the final pseudochromosomal assembly with NextGenMap and filtered for proper pairs. We generated a pileup file (Samtools v.1.0, Li 2011) and used PoPoolation (v.1.2.2, Kofler et al. 2011) to filter insertions and deletions before calculating nucleotide diversity (*π*) over 50,000-bp windows in each pseudochromosomal contig.

## Results and Discussion

### ONT Assembly

**Fig. 1.**
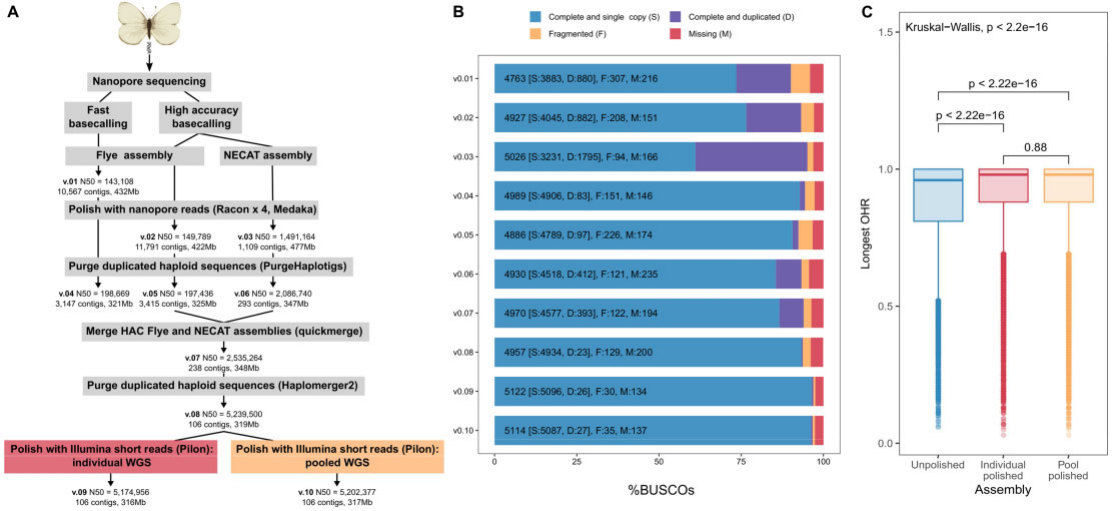
Genome assembly pipeline and metrics for 10 genomes show dramatic improvement during refinement steps. (*A*) Progressive increase in N50 and decrease in total contigs during polishing and merging of the nanopore assembly. Steps to refine the assembly included polishing with Illumina whole genome short reads (WGS) from a single individual and a pool of 18 individuals. (*B*) Assessment of the content and quality of 5,286 lepidopteran single copy orthologs shows complete, duplicated, fragmented or missing BUSCOs across the 10 assemblies (v.01–v.10). (*C*) Assessment of changes in genome quality using whole genome annotations shows similar effects of polishing using individual or PoolSeq Illumina data. (*C*) Polishing with Illumina short reads improved the ortholog hit ratio (OHR, values closer to 1 indicate a higher quality annotation).

Using only high-coverage (∼90×) ONT long reads, we achieved a highly contiguous *P. macdunnoughii* draft assembly (v0.08) totaling 319,093,312 bases across 106 contigs with an N50 of 5.20 Mb (fig. 1*A* and *B*; supplementary tables S1 and S2, Supplementary Material online). The assembly pipeline involved merging two long-read assemblies, one constructed with FLYE, the other with NECAT, each extensively polished and purged of duplicated haplotypes both before and after merging (v0.01–v0.08; details available in supplementary table S1, Supplementary Material online). Prior to polishing with additional short-read data, the v.0.08 draft assembly was already very complete, with 93.8% complete single copy Lepidoptera BUSCOs, with only 0.4% duplicated (fig. 1*B*).

### Short-read Polishing and Annotation Assessment

Illumina short reads from a single *P. macdunnoughii* female and from a pool of *P. macdunnoughii* females were both sufficient for polishing the v0.08 draft assembly, improving the assembly in comparable ways (fig. 1*A*–*C*; supplementary table S2, Supplementary Material online). Polishing increased complete lepidopteran BUSCOs to 96.9% in the individual-polished (v0.09) and 96.7% in the pool-polished (v0.10) assemblies.

Our annotation identified 19,640 good transcripts (containing start and stop codons) in the unpolished assembly, representing 17,362 unique genes (table 1). Annotations of the individual- and pool-polished genomes identified fewer transcripts and genes (18,347 and 18,603 good transcripts, respectively). To evaluate the annotations’ completeness, we compared them with a high-quality published annotation for *B. mori* (Bombycidae) using an OHR analysis (modified from O'Neil et al. 2010). This analysis compares putative orthologs in two annotations (e.g., *B. mori* proteins vs. unpolished *P. macdunnoughii* proteins) by generating a ratio of the *P. macdunnoughii* protein length to the *B. mori* protein length (ratios approaching 1 indicate more complete recovery of the expected ortholog length). Illumina polishing improved the number and OHR of recovered orthologs in the *P. macdunnoughii* annotations, as expected (Miller et al. 2018), regardless of whether individual or PoolSeq reads were used (fig. 1*C*; table 1).

**Table 1:** Genes Identified in Braker2 Annotations of *Pieris macdunnoughii* Assemblies and Ortholog Hit Ratio (OHR) Analysis with *Bombyx mori*

	Annotation			
	*B. mori*	Unpolished	Individual Polished	Pool Polished
Good transcripts	NA	19,640	18,347	18,603
Genes	14,802	17,362	16,251	16,496
Clustered proteins (90%)	14,439	17,550	16,260	16,501
Total *B. mori* orthologs	NA	13,599	13,637	13,669
Median OHR of longest hit	NA	0.96	0.98	0.98
Longest hits with OHR > 0.95	NA	7,062	8,203	8,233
Median identity of longest hit	NA	63.1%	64.6%	64.6%
Longest hits with identity > 95%	NA	240	277	283
Hits in both the unpolished and polished annotation	NA	NA	13,524	13,550

Note.—NA, not applicable.

### Synteny and Z Chromosome Validation

There was a high degree of chromosome-scale synteny between the pool-polished assembly (v0.10) and the *P. napi* reference genome, validating our assembly accuracy at this scale. This synteny corroborates a massive chromosomal rearrangement in *P. napi* relative to model lepidopteran systems with chromosome-level assemblies, as previously reported by Hill et al. (2019). Combined with evidence that this rearrangement is also shared by the cabbage white butterfly *Pieris rapae* (Hill et al. 2019), our results suggest that despite the rampant rate of chromosomal rearrangements in the ancestral lineage of *Pieris* butterflies, no major fissions or fusions have subsequently occurred.

**Fig. 2.**
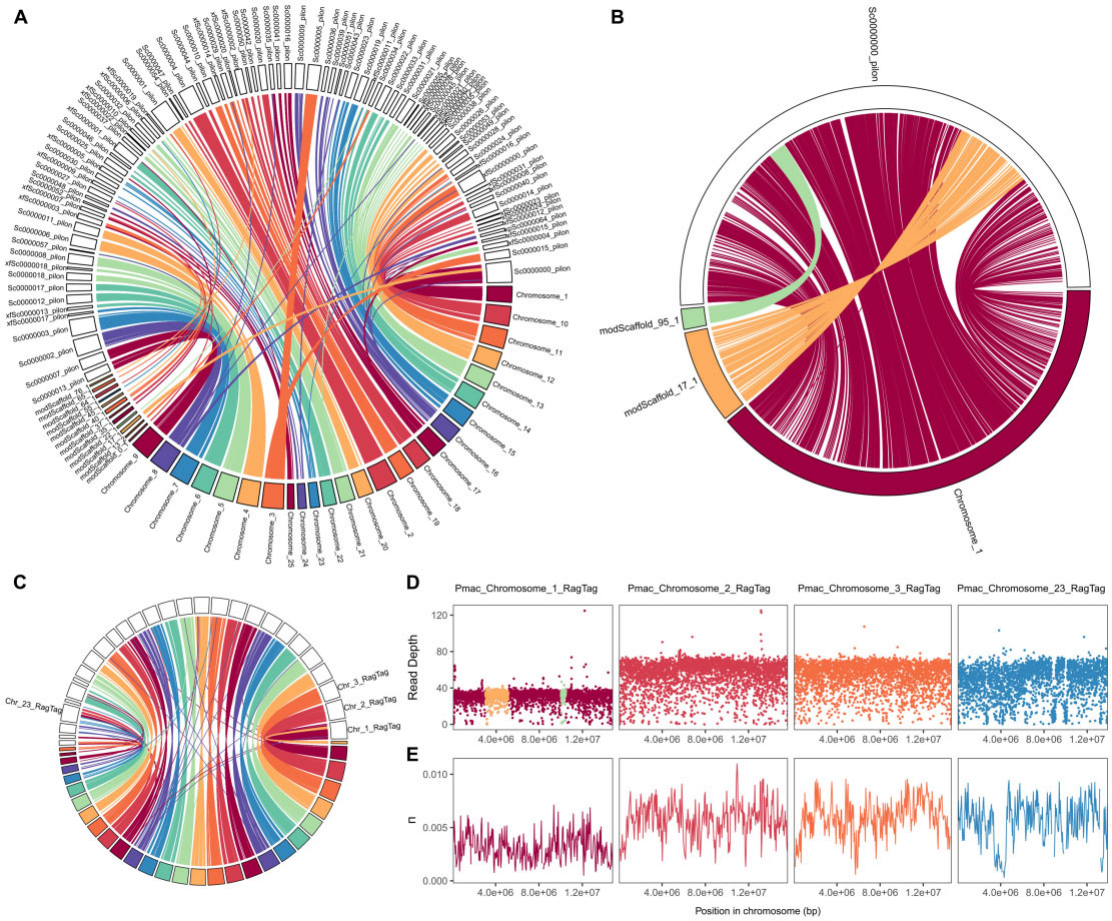
Chromosome-level assessment of synteny, read depth and genetic variation in *Pieris macdunnoughii.* (*A*) A circle plot showing each contig of the *P. macdunnoughii* v0.10 (noncolored scaffolds) and *Pieris napi* (colored scaffolds, showing scaffolds > 1 Mb representing 90.9% of the 318 bp assembly) assemblies, with lines between them showing aligned genomic regions of >5000 bp and 90% identity (for example, *P. napi* Chromosome 2 is covered by four *P. macdunnoughii* scaffolds, Sc0000044, Sc0000004, Sc0000047, and Sc0000054). (*B*) Detailed assessment of the alignments between the Z chromosome of *P. macdunnoughii* (Sc0000000) and aligned, unplaced *P. napi* scaffolds modScaffold_17_1 and modScaffold_95_1, supporting their inclusion on the Z chromosome between 3003162 and 5135852 bp and 10002530–10466150 bp, respectively. (*C*) The final pseudo-chromosomal *P. macdunnoughii* assembly (v0.10_RagTag) aligned with *P. napi.* (*D*) Consistently lower read depths of PoolSeq reads mapped to the pseudochromosomal assembly support conclusions about the Z chromosome (Pmac_chromosome_1_RagTag; colors as in B). (*E*) Nucleotide diversity (*π*), varied across the genome, as seen in representative autosomes 2, 3, and 23.

Extensive synteny supported superscaffolding the *P. macdunnoughii* genome using the *P. napi* chromosomes, thereby producing what we refer to as a pseudochromosomal assembly (v0.10_RagTag), which reduced our assembly to 47 contigs with an N50 of 13.0 Mb (fig. 2*C*; supplementary table S2, Supplementary Material online). Of the 106 contigs of the *P. macdunnoughii* assembly, 84 (288,535,466 bp, 91.1%) were aligned to chromosomes 1–25 of the *P. napi* assembly, whereas 22 contigs (28,013,828 bp) remained unplaced.

Although previous work suggested that two unplaced *P. napi* scaffolds belonged on the Z chromosome (Pruisscher et al. 2021), our assembly of *P. macdunnoughii* indicates where they should be located (fig. 2*B*). We validated that *P. macdunnoughii* Sc0000000/Chromsome_1_RagTag is a contiguous Z chromosome by assessing the depth of PoolSeq reads across this chromosome, finding half as many reads mapped (30.2 ± 0.3) compared with the autosomes (51.2 ± 0.4; fig. 2*D*), consistent with females having only one Z chromosome. We then used our PoolSeq data to estimate nucleotide diversity (*π*), calculated across 50kbp sliding windows, identifying 4,876,412 variable sites, and estimating a genome-wide mean for autosomal *π* of 0.0060. Genetic diversity was on average 52.3% lower on the Z chromosome (Chromsome_1_RagTag) than on the autosomes. Across autosomes, estimates of *π* were relatively even and similar (e.g., Chromosome_2_RagTag and Chromosome_3_RagTag, fig. 2*E*), though we identified several regions of low genetic diversity (e.g., Chromosome_23). This analysis illustrates the dual potential of PoolSeq data in a genome assembly pipeline, both as an effective data set for short-read polishing and resource for population genetic inferences.

## Conclusion

Our assembly for *P. macdunnoughii*, an emerging model system for studying maladaptation in plant–insect coevolutionary interactions, placed 91.1% of the 317 Mb genome into a chromosomal framework and exhibited a high and accurate gene content using BUSCO metrics. We annotated 18,603 good transcripts for 16,496 genes. Our results provide an important validation of previously reported chromosomal rearrangements in *Pieris* butterflies and insights into their Z chromosome evolution, not only supporting the neo-Z structure of *P. napi* but indicating that this fusion event happened before the split between European and North American lineages of *P. napi*. Further, we localized two large, previously unplaced scaffolds of *P. napi* into their proper locations on the Z chromosome. This genome assembly and its functional annotation lay the groundwork for identifying the genetic basis of persistent maladaptive egg-laying behavior by *P. macdunnoughii*, as well as the inability of larvae to eat invasive Eurasian mustards. At least two of the other North American *Pieris* species are involved in similar maladaptive host–plant interactions (Keeler and Chew 2008; Davis and Cipollini 2014). This genome will serve as an important resource for future research into susceptibility and responses to evolutionary traps.

## Supplementary Material

Supplementary data are available at *Genome Biology and Evolution* online.

## Supplementary Material

evab053_Supplementary_Data
