# Supplementary material for: The Genome of the Margined White Butterfly (Pieris macdunnoughii): Sex Chromosome Insights and the Power of Polishing with PoolSeq Data
Source: Genome Biol Evol. 2021 Mar 19;13(4):evab053. doi: 10.1093/gbe/evab053 (PMC8085124; doi:10.1093/gbe/evab053)
Supplement: evab053_Supplementary_Data [file evab053_supplementary_data.zip › Pmac_genome_MS_supplTablesFigs.docx]

Table S1. Assembly information for *Pieris macdunnoughii* v0.01 to 0.10_RagTag.

| **Version** | **Guppy Basecalling** | **Assembler** | **Long read polishing^1^** | **Purgehaplotigs** | **Quickmerge** | **Haplomerger2** | **Short read polishing^2^** | **RagTag alignment** |
| --- | --- | --- | --- | --- | --- | --- | --- | --- |
| **v0.01** | Fast | Flye | N | N | N | N | N | NA |
| **v0.02** | High accuracy | Flye | Y | N | N | N | N | NA |
| **v0.03** | High accuracy | NECAT | Y | N | N | N | N | NA |
| **v0.04** | Fast | Flye | Y | Y | N | N | N | NA |
| **v0.05** | High accuracy | Flye | Y | Y | N | N | N | NA |
| **v0.06** | High accuracy | NECAT | Y | Y | N | N | N | NA |
| **v0.07** | High accuracy | Flye+NECAT | Y | Y | Y | N | N | NA |
| **v0.08** | High accuracy | Flye+NECAT | Y | Y | Y | Y | N | NA |
| **v0.09** | High accuracy | Flye+NECAT | Y | Y | Y | Y | Individual (84X) | NA |
| **NA** | High accuracy | Flye+NECAT | Y | Y | Y | Y | Individual (68X) | NA |
| **v0.10** | High accuracy | Flye+NECAT | Y | Y | Y | Y | Pool (67X) | NA |
| **v0.10 RagTag** | High accuracy | Flye+NECAT | Y | Y | Y | Y | Pool (67X) | *Pieris napi* chromosomes, >80% identity |

^1^Rakon x4, Medaka x1

^2^Pilon

Table S2. Assembly statistics for *Pieris macdunnoughii* final assemblies (v0.10 v0.10_RagTag, the pseudochromosomal assembly made from aligning v0.10 with the *Pieris napi* genome), and preliminary assemblies v0.01-v0.09. Differences among the assemblies can be found in Table S2. BUSCOs^1^ were assessed with the Lepidoptera ODB 10 with a total of 5286 single-copy orthologs.

|  | **Version** | **Contigs** | **Total length** | **N50** | **Min. contig length** | **Avg. contig length** | **Max. contig length** | **Masked Repeats** | **BUSCOs^1^** | | | | |
| --- | --- | --- | --- | --- | --- | --- | --- | --- | --- | --- | --- | --- | --- |
|  |  |  |  |  |  |  |  |  | **C** | **S** | **D** | **F** | **M** |
| **Reported genome assemblies** | v0.10 | 106 | 316549294 | 5202377 | 644 | 2986314 | 14824368 | 35.03% | 5114 | 5087 | 27 | 35 | 137 |
|  | v0.10 RagTag | 47 | 316555194 | 12956643 | 644 | 6735217 | 15650382 | NA | 5116 | 5091 | 25 | 35 | 135 |
| **Preliminary assemblies** | v0.01 | 10567 | 431942183 | 143108 | 15 | 40877 | 8259670 | NA | 4763 | 3883 | 880 | 307 | 216 |
|  | v0.02 | 11791 | 421858107 | 149789 | 66 | 35778 | 8295014 | NA | 4927 | 4045 | 882 | 208 | 151 |
|  | v0.03 | 1109 | 477314139 | 1491164 | 82 | 430401 | 6456938 | NA | 5026 | 3231 | 1795 | 94 | 166 |
|  | v0.04 | 3147 | 320914943 | 198669 | 46 | 101975 | 8281812 | NA | 4989 | 4906 | 83 | 151 | 146 |
|  | v0.05 | 3415 | 324675409 | 197436 | 67 | 95073 | 8295014 | NA | 4886 | 4789 | 97 | 226 | 174 |
|  | v0.06 | 293 | 346761953 | 2086740 | 125 | 1183488 | 6456938 | NA | 4930 | 4518 | 412 | 121 | 235 |
|  | v0.07 | 238 | 348220470 | 2535264 | 125 | 1463111 | 14659823 | NA | 4970 | 4577 | 393 | 122 | 194 |
|  | v0.08 | 106 | 319093312 | 5239500 | 644 | 3010314 | 14890372 | 35.21% | 4956 | 4934 | 23 | 129 | 200 |
|  | v0.09 | 106 | 316067271 | 5174956 | 644 | 2981767 | 14783675 | 34.93% | 5122 | 5096 | 26 | 30 | 134 |
|  | NA | 106 | 315993601 | 5170597 | 644 | 2981072 | 14778052 | NA | 5116 | 5089 | 27 | 35 | 135 |

^1^ Benchmarking Universal Single-Copy Orthologs; C = complete; S = complete, single-copy; D = complete, duplicated; F = fragmented; M = missing

Table S3: Cleaning and filtering statistics for Illumina short reads.

| **Reads** | **Raw Reads (pairs)** | **Clones filtered (pairs)** | **Clones removed (percent)** | **Filtered reads** | **Filtered bases** | **Adapter trimmed reads** | **Adapter trimmed reads (percent)** | **Adapter trimmed bases** | **Adapter trimmed bases (percent)** | **Quality trimmed reads** | **Quality trimmed reads (percent)** | **Quality trimmed bases** | **Quality trimmed bases (percent)** |
| --- | --- | --- | --- | --- | --- | --- | --- | --- | --- | --- | --- | --- | --- |
| **Pmac_Mums (Pool)** | 9.26E+07 | 8.99E+07 | 2.86% | 1.80E+08 | 2.72E+10 | 1.78E+08 | 99.05% | 2.69E+10 | 98.91% | 1.63E+08 | 91.59% | 2.24E+10 | 83.35% |
| **Pmac000.1 (Individual)** | 1.12E+08 | 1.06E+08 | 5.44% | 2.13E+08 | 3.21E+10 | 2.12E+08 | 99.80% | 3.19E+10 | 99.42% | 1.98E+08 | 93.17% | 2.84E+10 | 88.97% |

Table S4. Mapping statistics for Illumina short reads.

| **Illumina short-read data** | **Assembly** | **Reads mapped** | **Percent reads mapped** | **Est. Insert size** | **Coverage** | **Insert mean** | **Insert SD** | **Percent reads in pairs** | **Read length** |
| --- | --- | --- | --- | --- | --- | --- | --- | --- | --- |
| **Poolseq reads (Pmac Mums)** | v0.08 | 155202183 | 95.12 | 532 | 66.69 | 256.36 | 145.72 | 87.8 | 151 |
| **Individual reads (Pmac000.1)** | v0.08 | 187864861 | 95.02 | 355 | 84.47 | 67.63 | 122.82 | 89.9 | 151 |
| **Subsampled individual reads (Pmac000.1)** | v0.08 | 152030311 | 95.02 | 355 | 68.38 | 67.75 | 123.18 | 90.2 | 151 |
| **Poolseq reads (Pmac Mums)** | v0.10_RagTag | 155667598 | 95.41 | 532 | 59.63 | 255.52 | 145.23 | 83.8 | 151 |
